# Whole-Genome Sequence of “*Candidatus* Liberibacter asiaticus” from Guangdong, China

**DOI:** 10.1128/genomeA.00273-14

**Published:** 2014-04-10

**Authors:** Z. Zheng, X. Deng, J. Chen

**Affiliations:** a Department of Plant Pathology, South China Agricultural University, Guangzhou, Guangdong, China; b USDA-ARS, San Joaquín Valley Agricultural Sciences Center, Parlier, California, USA

## Abstract

The draft genome sequence of “*Candidatus* Liberibacter asiaticus” strain A4, isolated from a mandarin citrus in Guangdong, People’s Republic of China, is reported. The A4 strain has a genome size of 1,208,625 bp, G+C content of 36.4%, 1,107 predicted open reading frames, and 53 RNA genes.

## GENOME ANNOUNCEMENT

The organism “*Candidatus* Liberibacter asiaticus” is an unculturable alphaproteobacterium associated with citrus huanglongbing (HLB) (yellow shoot disease), a devastating disease in citrus production worldwide (1–3). In China, HLB has been observed for over a hundred years in Guangdong Province (3). The association of “*Candidatus* Liberibacter asiaticus” to HLB was not established until 1996 (4, 5). Knowledge about “*Candidatus* Liberibacter asiaticus” is critical for HLB control. Due to the lack of *in vitro* culture, little is known about the bacterium, particularly differences among strains of various geographical origins. Whole-genome sequencing has generated a significant amount of information, which has facilitated the biological characterization of this bacterium (6, 7). Here, we report a draft whole-genome sequence of “*Candidatus* Liberibacter asiaticus” from Guangdong.

“*Candidatus* Liberibacter asiaticus” strain A4 was established, maintained, and enriched in periwinkle [*Catharanthus roseus* (L.) G. Don.] in an insect-proof screen house on the campus of the South China Agricultural University in Guangzhou, China. The strain was originally collected from an HLB mandarin citrus (*Citrus reticulata* cultivar Shatangju) in Sihui City (23°25′14″N, 112°32′04″E) of Guangdong Province and was transmitted to a healthy Shatangju plant through grafting in 2005. In 2007, “*Candidatus* Liberibacter asiaticus” was transmitted from citrus to periwinkle via dodder (*Cuscuta campestris* Yunck). For sequencing, DNA was extracted from infected periwinkle tissue using the cetyltrimethylammonium bromide (CTAB) method as previously described (8, 9). Bacterial DNA was further enriched using an NEBNext Microbiome DNA enrichment kit (New England BioLabs, Inc., Ipswich, MA), amplified through a REPLI-g minikit (Qiagen, Inc., Valencia, CA), and sequenced using Illumina MiSeq (Illumina, Inc., San Diego, CA).

A total of 3.28 × 10^7^ reads (8.16 × 10^9^ bp) with a mean of 249 bp per read were generated from the “*Candidatus* Liberibacter asiaticus”-enriched DNA sample. Using the whole-genome sequence of “*Candidatus* Liberibacter asiaticus” strain Psy62 (6) as a reference, a total of 721,209 reads were identified using standalone BLAST software (10). The “*Candidatus* Liberibacter asiaticus” reads were collected using a Perl script and assembled using CLC Genomics Workbench 7.0 software that generated 11 contigs ranging from 287 bp to 556,195 bp, which achieved ~143× coverage. The draft genome of “*Candidatus* Liberibacter asiaticus” strain A4 comprises 1,208,625 bp, with the G+C content of 36.4%. Annotation was performed by the RAST server (http://rast.nmpdr.org/) (11). The A4 genome was predicted to have 1,107 open reading frames (ORFs) and 53 RNA genes.

The A4 draft genome is 98.5% of the Psy62 genome (1,227,204 bp) (6) and 95.3% of the GX-1 genome (1,268,237 bp) (7). Different from the Psy62 and GX-1 genome sequences of psyllid origins, the A4 genome sequence originated from a plant source, which could be used to study bacterial genome variation among different habitats (insect vector versus plant host).

### Nucleotide sequence accession number.

This whole-genome shotgun project has been deposited at DDBJ/EMBL/GenBank under the accession number JFGQ00000000.
